# CCKBR (Gastrin Receptor) as a Theranostic Target in Oncology: Biological Rationale, Diagnostic Imaging, and Next-Generation Peptide-Based Drug Development

**DOI:** 10.3390/pharmaceutics18101198

**Published:** 2026-09-22

**Authors:** Ming-Cheng Chang, Chun-Tang Chen, Wan-Chi Lee, Yi-Jou Tai, Ying-Cheng Chiang

**Affiliations:** 1 Department of Isotope Application Research, National Atomic Research Institute, No. 1000, Wenhua Rd., Longtan Dist., Taoyuan City 325207, Taiwan; mcchang@nari.org.tw (M.-C.C.); ctchen@nari.org.tw (C.-T.C.); leewc@nari.org.tw (W.-C.L.); 2 Department of Obstetrics and Gynecology, College of Medicine, National Taiwan University, Taipei 100226, Taiwan; 3 Department of Obstetrics and Gynecology, National Taiwan University Hospital, Taipei 100226, Taiwan; 4 Department of Obstetrics and Gynecology, National Taiwan University Hospital Hsin-Chu Branch, Hsinchu City 300195, Taiwan

**Keywords:** CCKBR, theranostics, radioligand therapy, molecular imaging, precision oncology

## Abstract

Cholecystokinin B receptor (CCKBR) is a promising theranostic target because of its overexpression in several malignancies, including medullary thyroid carcinoma, small-cell lung cancer, gastric cancer, and pancreatic cancer. Its high-affinity ligand binding and efficient receptor-mediated internalization provide a strong biological basis for molecular imaging and peptide receptor radionuclide therapy (PRRT). Recent advances in radiolabeled gastrin and mini-gastrin analogues have improved tumor targeting, metabolic stability, and pharmacokinetic profiles, supporting their application in both diagnostic imaging and radionuclide therapy. This review summarizes the molecular biology and tumor expression of CCKBR, recent progress in SPECT/PET imaging and targeted radionuclide therapy, and advances in ligand and pharmacokinetic engineering. Despite substantial progress, CCKBR-targeted theranostics remain less clinically established than SSTR- or PSMA-based platforms. Major challenges include heterogeneous receptor expression, renal and gastric uptake, limited prospective clinical evidence, and the lack of standardized imaging, dosimetry, and treatment protocols. Addressing these limitations will be essential for the broader clinical translation of CCKBR-targeted theranostics.

## 1. Introduction

Precision oncology has increasingly shifted toward molecularly defined, receptor-mediated therapeutic strategies that enable selective delivery of diagnostic and therapeutic payloads to malignant cells while minimizing exposure to normal tissues. A major unmet clinical need remains the identification of tumor-associated targets that combine high tumor specificity, efficient receptor-mediated internalization, and prolonged intratumoral retention, properties that are critical for both molecular imaging and targeted radionuclide therapy [1,2]. Among druggable protein families, G protein-coupled receptors (GPCRs) constitute one of the most versatile platforms for oncology theranostics because of their membrane accessibility, signal amplification capacity, and ligand-dependent receptor trafficking characteristics [3,4]. Clinically successful examples include somatostatin receptor-targeted imaging and peptide receptor radionuclide therapy (PRRT), exemplified by ^177^Lu-DOTATATE (LUTATHERA^®^), the first FDA-approved radiolabeled GPCR-targeting therapeutic agent and a landmark achievement in receptor-guided precision oncology [5].

Within this framework, the cholecystokinin B receptor (CCKBR) has emerged as a promising theranostic target; however, compared with well-established SSTR- and PSMA-directed theranostic platforms, its clinical translation remains at a relatively early stage [6]. Unlike many GPCRs that are either physiologically restricted or broadly distributed in normal tissues, CCKBR exhibits high receptor density in selected malignancies, including gastric cancer, pancreatic cancer, and neuroendocrine tumors such as medullary thyroid carcinoma [6,7,8]. Furthermore, its classification as a Class A rhodopsin-like GPCR and its favorable ligand-dependent trafficking behavior provide a biologically attractive platform for the development of peptide-based imaging probes and targeted therapeutic constructs. These characteristics support both high-contrast molecular imaging and efficient intracellular delivery of therapeutic payloads.

Over the past few years, CCKBR-targeted theranostics has entered a new phase of clinical translation. Prospective clinical studies of the mini-gastrin analogue CP04 have established the feasibility of receptor-targeted imaging in medullary thyroid carcinoma (MTC), while early investigations of ^177^Lu-labeled mini-gastrin analogues have demonstrated the therapeutic potential of peptide receptor radionuclide therapy [9,10]. Meanwhile, increasing evidence indicates that CCKBR expression extends beyond MTC to include gastric cancer, small-cell lung cancer (SCLC), and other receptor-positive malignancies, thereby broadening the potential clinical applications of CCKBR-directed imaging and therapy [11,12]. In parallel, advances in peptide engineering, radiopharmaceutical design, and quantitative molecular imaging have substantially improved tracer stability, pharmacokinetics, and patient stratification. These developments collectively highlight the rapid evolution of the field and underscore the need for an updated review that integrates recent clinical evidence with biological mechanisms, molecular imaging, targeted radionuclide therapy, and next-generation theranostic strategies.

Previous reviews have provided important foundations for CCKBR-targeted theranostics. Klingler et al. primarily emphasized the development, limitations, and optimization of radiolabeled CCK2R-targeting peptide analogues [10], whereas the more recent review by Li et al. provided a broader overview of CCK2R-directed cancer radiotheranostics and radiopharmaceutical development [13]. Building on these contributions, the present review adopts a comprehensive translational perspective by integrating five key aspects: (1) the biological rationale for CCKBR-targeted theranostics; (2) current evidence supporting molecular imaging for diagnosis, quantitative receptor assessment, and patient stratification; (3) recent progress and remaining evidence gaps in targeted radionuclide therapy; (4) next-generation peptide engineering, including scaffold optimization, pharmacokinetic modulation, and radiometal–chelator design; and (5) emerging technologies such as artificial intelligence-assisted imaging analysis and precision theranostic development. The added value of this review lies in integrating CCKBR biology, molecular engineering, imaging-based patient selection, and therapeutic delivery within a unified translational framework, while highlighting key barriers to clinical implementation.

### Materials and Methods

This article was prepared as a narrative review. Relevant literature was identified through searches of PubMed, Web of Science, and Scopus using combinations of keywords including “CCKBR”, “gastrin receptor”, “minigastrin”, “theranostics”, “molecular imaging”, “radioligand therapy”, “peptide receptor radionuclide therapy”, and related terms. Publications available up to March 2025 were considered. Priority was given to peer-reviewed original research articles, clinical studies, and representative review articles that contributed to the understanding of CCKBR biology, molecular imaging, radionuclide therapy, and peptide engineering. Literature selection emphasized studies with clear translational relevance, spanning receptor biology, molecular imaging, radionuclide therapy, peptide engineering, and emerging technologies relevant to CCKBR-directed theranostics. Generative AI was used solely for English-language editing and improving the clarity and readability of the manuscript. It was not used for study design, data generation, data analysis, interpretation of results, or formulation of scientific conclusions. All AI-assisted text was critically reviewed and revised by the authors.

## 2. Molecular Biology and Translational Significance of CCKBR

The cholecystokinin B receptor (CCKBR) is a member of the Class A rhodopsin-like G protein-coupled receptor (GPCR) family and plays important physiological roles in gastrointestinal and neuroendocrine regulation. Under normal conditions, CCKBR is predominantly expressed in the gastrointestinal tract and central nervous system; however, aberrant receptor overexpression has been reported in several malignancies, supporting its development as a potential molecular imaging and therapeutic target [14,15]. Beyond its physiological functions, CCKBR possesses several characteristics that are particularly relevant for oncologic theranostics, including membrane accessibility, ligand-dependent signaling, receptor-mediated endocytosis, and favorable intracellular trafficking behavior. The following sections summarize the structural features, signaling pathways, ligand interactions, and receptor trafficking mechanisms that contribute to the biological and translational significance of CCKBR.

### 2.1. Structural Features Relevant to Theranostics

CCKBR is a Class A rhodopsin-like GPCR characterized by a conserved seven-transmembrane architecture that enables high-affinity recognition of gastrin and cholecystokinin-derived peptides [7]. Unlike many intracellular therapeutic targets, CCKBR is located on the cell surface, providing direct accessibility for peptide-based imaging probes and radiopharmaceuticals [7,16,17]. Its restricted expression in normal tissues and overexpression in selected malignancies further support its suitability as a molecular target for cancer theranostics.

Beyond ligand recognition, one of the most clinically relevant features of CCKBR is its ability to undergo agonist-induced receptor internalization following ligand binding [16,18,19]. This process promotes intracellular accumulation and prolonged retention of receptor-bound agents within tumor cells, thereby enhancing imaging contrast and therapeutic radiation delivery [20]. These structural characteristics provide the biological foundation for the development of CCKBR-targeted molecular imaging and PRRT.

### 2.2. Tumor-Associated Signaling

Upon activation by gastrin or cholecystokinin-derived peptides, CCKBR primarily couples to Gq/11 proteins, leading to activation of the PLC/IP_3_/Ca^2+^ signaling cascade together with the MAPK/ERK and PI3K/AKT pathways [18,19,21]. Experimental studies in CCKBR-expressing cancer models have linked activation of these pathways to cellular proliferation, survival, migration, and differentiation, thereby contributing to tumor growth and disease progression in CCKBR-expressing malignancies [22].

From a theranostic perspective, these downstream pathways are more relevant for establishing the oncogenic significance of CCKBR than for directly determining radiopharmaceutical uptake. For molecular imaging, receptor abundance, membrane accessibility, and high-affinity ligand binding are the principal determinants of target visualization. In therapeutic applications, efficient agonist-induced internalization and prolonged intracellular retention are particularly important because they increase tumor residence time and radiation delivery [23]. Thus, CCKBR signaling supports the biological rationale for targeting receptor-positive tumors [7]. Receptor accessibility and ligand binding are more directly relevant to molecular imaging, whereas receptor internalization and intracellular retention are particularly important for therapeutic radionuclide delivery.

### 2.3. Ligand Recognition and Peptide Design

CCKBR is physiologically activated by endogenous peptide ligands, including gastrin, CCK-8, and CCK-4, all of which share a conserved C-terminal amidated sequence required for receptor recognition [24,25]. Although these ligands differ in peptide length, sulfation, and biological activity, their common pharmacophore provides the structural basis for receptor binding. Among them, gastrin has become the principal template for radiopharmaceutical development because its C-terminal binding motif can be preserved while allowing extensive modification of the N-terminal region [26,27].

This structural flexibility has led to the development of mini-gastrin analogues, which retain high receptor affinity while exhibiting improved metabolic stability and greater compatibility with molecular engineering. Most CCKBR-targeted tracers are further conjugated with the macrocyclic chelator DOTA, enabling stable labeling with diagnostic radionuclides such as ^68^Ga and therapeutic radionuclides such as ^177^Lu using the same peptide scaffold. In addition, agonist ligands are generally preferred because receptor activation triggers internalization, resulting in prolonged intracellular retention of radiolabeled peptides within tumor cells [8,28]. For diagnostic imaging, ligand designs favoring rapid blood clearance and high tumor-to-background contrast are preferable, whereas therapeutic applications place greater emphasis on prolonged tumor retention, efficient internalization, and a favorable tumor-to-organ dose ratio. Thus, the optimal ligand profile may differ according to whether the primary goal is lesion visualization or sustained radionuclide delivery. Consequently, agonist-based mini-gastrin analogues combined with DOTA chemistry currently represent the most versatile platform for CCKBR-targeted imaging and PRRT, providing the molecular foundation for the clinical applications discussed in the following sections [29,30].

### 2.4. Receptor Internalization and Tumor Retention

One of the defining biological features that distinguishes CCKBR from many other molecular targets is its efficient agonist-induced receptor internalization [19]. Following ligand binding, activated CCKBR is rapidly internalized through a β-arrestin-dependent, clathrin-mediated pathway, allowing receptor–ligand complexes to enter the intracellular compartment (Figure 1). Rather than representing only a mechanism of receptor regulation, this process has important translational implications because it enables intracellular delivery of radiolabeled peptides and therapeutic payloads.

After internalization, receptor–ligand complexes are transported through endosomal compartments before being directed either toward lysosomal degradation or recycling back to the cell surface. From a theranostic perspective, the precise trafficking pathway is less important than its overall consequence: prolonged intracellular retention of receptor-bound agents [19,31]. Unlike ligands that remain confined to the plasma membrane, internalized radiopharmaceuticals are retained within tumor cells for extended periods, thereby increasing tumor residence time and improving target-to-background contrast. This property is particularly advantageous for molecular imaging, where sustained tracer accumulation enhances lesion visualization, and for radionuclide therapy, where prolonged retention increases radiation delivery to receptor-positive tumors.

The ability of CCKBR to undergo efficient receptor-mediated internalization also provides a strong biological rationale for PRRT. Therapeutic radionuclides such as ^177^Lu rely on sufficient intracellular residence to maximize radiation dose deposition within tumor tissue [8,12,13,17,32]. Consequently, receptor internalization has become a key determinant of therapeutic efficacy, making agonist ligands preferable to non-internalizing alternatives for most CCKBR-targeted applications.

Recognition of this biological property has profoundly influenced radiopharmaceutical development. Current ligand engineering strategies are designed not only to preserve high receptor affinity but also to enhance intracellular retention while improving metabolic stability, pharmacokinetics, and tumor-to-organ uptake ratios. Structural optimization of mini-gastrin analogues, linker modification, albumin-binding approaches, and renal-protective strategies have all been developed with the common goal of maximizing tumor retention without increasing off-target radiation exposure [8,33,34]. Collectively, receptor internalization and prolonged intracellular retention provide the mechanistic foundation for CCKBR-targeted theranostics and continue to guide the development of next-generation imaging agents and therapeutic radiopharmaceuticals.

### 2.5. Clinical Implications of CCKBR Biology

The biological characteristics of CCKBR provide a strong mechanistic foundation for its application in precision theranostics. Unlike many molecular targets that merely exhibit tumor-associated expression, CCKBR combines several favorable properties, including cell-surface accessibility, high-affinity ligand recognition, efficient receptor-mediated internalization, and prolonged intracellular retention [35,36]. Collectively, these features enable selective delivery of diagnostic and therapeutic agents to receptor-positive tumors while minimizing exposure to surrounding normal tissues. From a diagnostic perspective, high receptor density and sustained membrane expression facilitate high-contrast molecular imaging using radiolabeled mini-gastrin analogues. Quantitative assessment of receptor expression can support patient selection, evaluate whole-body disease burden, and provide individualized information for treatment planning [37,38]. These imaging characteristics also establish the biological basis for theranostic pairing, in which structurally related ligands are used for both diagnostic imaging and targeted radionuclide therapy. For therapeutic applications, receptor-mediated internalization is particularly advantageous because it prolongs the intracellular retention of therapeutic radionuclides, thereby increasing radiation delivery to tumor cells. This biological property has guided the development of agonist-based mini-gastrin analogues and continues to influence strategies for optimizing peptide stability, pharmacokinetics, tumor retention, and kidney protection. Together, these translational advances demonstrate how fundamental knowledge of CCKBR biology has directly shaped radiopharmaceutical design and clinical implementation, supporting the continued development of next-generation CCKBR-targeted theranostic agents.

## 3. CCKBR Expression Heterogeneity and Implications for Targeted Theranostics

The clinical success of receptor-directed theranostics depends not only on the presence of a molecular target but also on its expression level, spatial distribution, and retention characteristics within tumors and normal tissues. CCKBR demonstrates selective enrichment in several neuroendocrine and epithelial malignancies while remaining relatively restricted in most peripheral organs, providing a favorable biological basis for receptor-targeted imaging and radionuclide therapy [20,39]. However, receptor expression is heterogeneous across tumor types and individual lesions, and these variations may influence ligand uptake and therapeutic response [40,41] (Figure 2). In addition to tumor heterogeneity, physiologic CCKBR expression introduces important translational considerations. Gastric enterochromaffin-like cells represent a major physiologic site of receptor expression [42,43], whereas renal tubular peptide handling contributes substantially to kidney uptake of radiolabeled gastrin analogs [32,44]. These tissues therefore constitute important physiologic dose-limiting organs that must be considered during dosimetric evaluation and therapeutic development. Nevertheless, these physiologic uptake sites also provide opportunities for quantitative dosimetry assessment and rational ligand engineering aimed at improving tumor-to-organ therapeutic indices.

From a clinical perspective, CCKBR heterogeneity may directly influence both patient selection and treatment outcome. Interpatient and interlesional differences in receptor expression can result in variable radiotracer uptake, making whole-body receptor imaging particularly important for identifying patients and lesions with sufficient target expression for CCKBR-directed therapy [13,45]. Moreover, intratumoral heterogeneity may lead to nonuniform radiopharmaceutical distribution and radiation dose deposition, potentially contributing to incomplete tumor control or heterogeneous treatment responses [46]. Quantitative molecular imaging therefore has the potential to serve not only as a diagnostic tool but also as a biomarker for treatment eligibility, lesion-level response prediction, and individualized dosimetry. Recognition of these heterogeneous expression patterns may improve clinical decision-making by enabling selection of patients most likely to benefit from CCKBR-targeted theranostics while avoiding treatment in patients with insufficient or highly discordant receptor expression [13,47,48].

Compared with established theranostic targets such as somatostatin receptors (SSTRs) and prostate-specific membrane antigen (PSMA), CCKBR shares several favorable characteristics, including cell-surface accessibility, high-affinity ligand binding, and the capacity for receptor-mediated internalization. However, important differences remain. SSTR- and PSMA-directed platforms benefit from extensive clinical validation, standardized imaging-based patient selection, and established therapeutic pathways, whereas CCKBR-targeted theranostics remain at an earlier stage of clinical translation. In addition, heterogeneous CCKBR expression across and within tumor types may complicate patient selection, while physiologic gastric expression and renal peptide handling can limit tumor-to-organ contrast and therapeutic index [13]. Conversely, the restricted tumor spectrum and internalizing properties of CCKBR may provide opportunities for highly selected receptor-positive populations [45]. Thus, CCKBR should currently be viewed as a complementary emerging target rather than a replacement for established SSTR- or PSMA-directed theranostic platforms.

### 3.1. Tumor Spectrum of CCKBR Expression

The therapeutic relevance of CCKBR targeting is largely associated with its preferential expression in malignancies characterized by neuroendocrine differentiation or gastrin-responsive signaling. Rather than being uniformly expressed across cancers, CCKBR expression reflects lineage-specific biology and signaling dependencies, providing a biologic basis for receptor-directed imaging and targeted therapeutic approaches.

Among the most consistently receptor-enriched malignancies are gastro-entero-pancreatic neuroendocrine tumors (Ks), which retain peptide hormone signaling pathways involved in secretion and cellular proliferation. Neuroendocrine tumors retain peptide hormone signaling pathways that provide a biologic rationale for receptor-directed imaging and therapeutic approaches, supporting efficient ligand accessibility and receptor targeting [49]. Similarly, medullary thyroid carcinoma, derived from parafollicular C cells, demonstrates high receptor positivity and favorable receptor trafficking characteristics, making it one of the earliest clinical candidates for CCKBR-directed imaging and peptide receptor radionuclide therapy [50].

Within epithelial malignancies, gastric adenocarcinoma and pancreatic ductal adenocarcinoma represent important translational targets. Gastrin signaling normally regulates gastric mucosal growth and homeostasis, and malignant transformation may amplify this pathway through receptor upregulation or autocrine stimulation loops [51,52]. Elevated CCKBR expression has been reported in subsets of gastric cancers and may contribute to gastrin-responsive tumor biology [52]. In pancreatic ductal adenocarcinoma, receptor-positive subsets appear enriched in gastrin-responsive cellular populations associated with invasive behavior and stromal interactions [53]. Although expression prevalence varies across cohorts, even moderate receptor density may provide opportunities for receptor-targeted therapeutic delivery.

Beyond the gastrointestinal tract, small-cell lung carcinoma and selected high-grade gliomas may further expand the potential clinical spectrum of CCKBR-targeted theranostics [54,55]. In small-cell lung carcinoma, successful visualization using CCKBR-targeted PET imaging suggests the presence of clinically relevant receptor expression that may support future theranostic applications [54]. Preliminary molecular evidence also suggests involvement of CCK-related signaling pathways in subsets of gliomas, although the therapeutic relevance of CCKBR-targeted approaches remains to be established [55]. Collectively, available evidence suggests that CCKBR expression is more closely associated with tumor signaling phenotype and lineage-specific biology than with anatomical origin alone, emphasizing the importance of receptor assessment for identifying tumors most likely to benefit from CCKBR-targeted strategies.

### 3.2. Receptor Heterogeneity and Theranostic Stratification

Recent transcriptomic studies, including single-cell RNA sequencing and spatial transcriptomic analyses, have begun to further demonstrate that CCKBR expression is spatially heterogeneous both within and across tumors [56,57]. These technologies reveal marked variability in receptor expression among tumor-cell subpopulations and the surrounding tumor microenvironment, suggesting that receptor availability may vary according to cellular phenotype, differentiation status, and local microenvironmental factors. Such heterogeneity may influence imaging sensitivity, therapeutic response, and patient selection, highlighting the importance of molecular imaging for non-invasive whole-body assessment of CCKBR expression. At present, no validated quantitative threshold of CCKBR expression has been established for treatment selection. Although PET studies have reported semiquantitative uptake parameters and immunohistochemical studies have evaluated receptor expression, neither an SUV-based cutoff nor a standardized IHC scoring threshold has been prospectively validated for predicting therapeutic benefit. This remains an important limitation and unmet need for clinical translation.

Intertumoral variability is particularly evident among gastrin-responsive and neuroendocrine-associated malignancies [58]. Neuroendocrine tumors and medullary thyroid carcinoma generally demonstrate high and relatively homogeneous membrane expression, whereas gastric and pancreatic malignancies exhibit greater variability in receptor expression across tumor subtypes and patient cohorts [58,59]. Receptor enrichment may localize to specific differentiation states, invasive fronts, or neuroendocrine-like subclones, resulting in substantial variability in target accessibility between tumors.

Intratumoral heterogeneity further complicates therapeutic delivery. Receptor expression may differ between proliferative regions, hypoxic niches, and stromal interfaces [60,61]. Inflammatory signaling, epithelial–mesenchymal transition, and microenvironmental stress have all been implicated in regulating GPCR expression [58]. Because receptor-mediated internalization contributes to intracellular retention of targeted agents, receptor-rich subclones may preferentially accumulate radionuclide or cytotoxic payloads, potentially contributing to heterogeneous treatment responses [62]. The recognition of intratumoral heterogeneity further supports the role of molecular imaging, as non-invasive whole-body imaging can characterize receptor expression across multiple lesions, overcoming the sampling limitations of conventional biopsy.

An additional challenge is temporal variability. Receptor expression may evolve during tumor progression or following systemic therapy through adaptive signaling rewiring and clonal selection [63,64]. Experimental evidence suggests that gastrin-associated signaling pathways can become amplified under therapeutic stress, potentially altering receptor availability over time [65]. These observations highlight the importance of real-time molecular imaging for treatment planning. Quantitative PET imaging enables noninvasive assessment of target expression and tumor-to-organ contrast immediately before therapy, providing an important tool for patient stratification in receptor-directed theranostics [66]. Understanding these spatial and temporal variations is therefore critical for optimizing treatment selection and predicting therapeutic retention in CCKBR-targeted theranostic applications.

### 3.3. Physiological Uptake and Dosimetric Constraints

Successful translation of CCKBR-directed theranostics requires consideration of physiologic receptor distribution in addition to tumor expression. In normal tissues, significant uptake is primarily confined to the gastric mucosa and renal tubular system, making these organs the principal determinants of off-target radiation exposure and dosimetric constraints [10,67].

Physiologic gastric uptake reflects endogenous gastrin signaling within enterochromaffin-like (ECL) cells of the fundic mucosa, where functional CCKBR expression regulates gastric acid secretion and mucosal homeostasis [68]. Consequently, peptide-based ligands demonstrate predictable receptor-mediated gastric accumulation following systemic administration. Gastric uptake is generally reproducible across individuals and therefore represents a predictable component of pre-treatment dosimetric assessment rather than an unpredictable source of toxicity [10,67].

In contrast, renal retention is largely receptor-independent and results from proximal tubular peptide reabsorption mechanisms [69]. Following glomerular filtration, radiolabeled peptides undergo megalin- and cubilin-mediated endocytosis within proximal tubular epithelial cells, leading to radionuclide retention in the renal cortex [33,70]. Because renal uptake is strongly influenced by peptide charge, linker composition, and molecular size, medicinal chemistry optimization has become an important strategy for reducing nephrotoxicity while preserving tumor-targeting efficiency [33].

Importantly, these physiologic uptake reservoirs do not preclude clinical application. Instead, they provide quantifiable parameters for individualized dosimetry and safety optimization. Amino acid co-infusion with lysine or arginine can reduce renal tubular reabsorption and lower kidney radiation exposure [71], while linker engineering, albumin-binding strategies, and charge redistribution may further improve tumor-to-organ contrast [72]. Together, these approaches support the development of CCKBR-targeted theranostics with improved therapeutic index and favorable safety profiles.

## 4. Clinical Translation of CCKBR-Targeted Molecular Imaging

Molecular imaging has become an essential component of modern theranostics, providing a noninvasive approach for target characterization, disease assessment, and treatment planning. In receptor-directed oncology, imaging serves not only as a diagnostic tool but also as a functional biomarker that enables real-time evaluation of target availability and tumor heterogeneity. For CCKBR-directed strategies, molecular imaging plays a central role in identifying receptor-positive disease and guiding subsequent therapeutic decision-making. Similar to the established SSTR imaging paradigm, CCKBR-targeted imaging aims to identify receptor-positive patients who are most likely to benefit from subsequent radionuclide therapy, although clinical validation remains considerably less mature.

Advances in peptide-based radiopharmaceutical design have enabled the development of highly specific CCKBR-targeted tracers for both SPECT and PET imaging. These agents allow noninvasive assessment of target expression, whole-body tumor burden, and tumor-to-organ contrast, thereby providing information that cannot be obtained from tissue-based assays alone [8,73]. In addition to lesion detection, imaging-derived metrics may support dosimetry estimation, treatment planning, and evaluation of target suitability before therapeutic intervention. Consequently, CCKBR-targeted imaging is increasingly integrated into theranostic workflows as a bridge between receptor biology and clinical application. This section reviews currently available imaging tracers, their diagnostic performance, less clinically established evidence for patient stratification, and the pharmacokinetic factors that influence image quality, tumor uptake, and treatment feasibility (Figure 3).

### 4.1. Mini-Gastrin and CCK-Based Imaging Tracers

The development of CCKBR-targeted molecular imaging has largely been based on peptide scaffolds derived from endogenous gastrin and cholecystokinin ligands, both of which retain the conserved C-terminal pharmacophore required for receptor recognition [74]. Early studies demonstrated that preservation of the Trp-Met-Asp-Phe-NH_2_ motif was sufficient to maintain high-affinity receptor binding, establishing mini-gastrin analogs as the principal platform for CCKBR-targeted radiopharmaceutical development [75].

Initial investigations employed gamma-emitting radionuclides for single-photon emission computed tomography [SPECT], demonstrating the feasibility of imaging receptor-positive neuroendocrine tumors and medullary thyroid carcinoma [10,67,76,77]. However, clinical translation was limited by rapid enzymatic degradation and substantial renal retention. Subsequent advances in peptide engineering and chelator chemistry enabled the development of positron emission tomography (PET) tracers, particularly gallium-68-labeled mini-gastrin analogs, which offered improved sensitivity, spatial resolution, and quantitative assessment of target expression across heterogeneous tumor lesions [73,77].

In parallel, cholecystokinin-derived ligands have been explored as alternative targeting scaffolds. Sulfated CCK analogs demonstrate high receptor affinity owing to favorable peptide–receptor interactions [78]. However, mini-gastrin–based tracers have generally shown more favorable tumor retention and broader compatibility with theranostic applications, particularly when combined with DOTA-based chelation systems [10,79]. Ongoing efforts continue to improve tracer stability, tumor uptake, and pharmacokinetic performance through peptide engineering and molecular optimization. Collectively, mini-gastrin and CCK-derived tracers form the foundation of CCKBR-targeted molecular imaging and enable noninvasive assessment of receptor expression for theranostic applications.

### 4.2. Diagnostic Performance and Patient-Stratification Utility of CCKBR-Targeted Imaging

The clinical value of CCKBR-targeted imaging depends not only on receptor recognition but also on its ability to accurately identify receptor-positive disease and support therapeutic decision-making. As theranostic strategies increasingly rely on imaging-guided treatment selection, diagnostic performance has become a critical determinant of clinical utility [13].

Early studies using SPECT-based mini-gastrin analogs demonstrated the feasibility of receptor-mediated imaging in neuroendocrine tumors and medullary thyroid carcinoma, providing lesion detection beyond that achievable with conventional anatomical imaging modalities [10,80]. However, limited spatial resolution and prolonged acquisition times restricted broader clinical implementation. The introduction of PET imaging substantially improved diagnostic performance. Gallium-68–labeled mini-gastrin analogs offer higher sensitivity, superior spatial resolution, and improved signal-to-noise characteristics, enabling more accurate detection of small lesions and comprehensive assessment of metastatic disease [28,29].

In addition to lesion detection, quantitative PET-derived parameters—including standardized uptake value (SUV), tumor-to-background ratio, and tumor-to-organ contrast—provide functional measures of receptor availability and target expression [81]. Whole-body imaging may also identify receptor-positive lesions that are not adequately represented by tissue sampling, highlighting an important advantage over biopsy-based assessment alone [13,28]. Although physiologic gastric and renal uptake generate predictable background activity, these patterns can generally be distinguished from malignant lesions based on their anatomical distribution and imaging kinetics [39,82].

A major clinical application of CCKBR-targeted imaging is patient stratification for theranostic interventions. Quantitative uptake metrics, particularly tumor-to-kidney and tumor-to-stomach contrast ratios, provide practical indicators of treatment suitability and dosimetric feasibility [7,80]. High tumor uptake and favorable tumor-to-organ contrast are generally considered desirable characteristics for therapeutic application and dosimetric optimization [83]. Consequently, CCKBR-targeted imaging functions not only as a diagnostic modality but also as a functional biomarker for treatment selection, individualized dosimetry, and risk–benefit assessment within receptor-directed therapeutic strategies [45].

### 4.3. Clinical Evidence and the Theranostic Pairing Paradigm in CCKBR-Targeted Imaging

The emergence of theranostic oncology has transformed molecular imaging from a diagnostic modality into a key component of treatment selection and therapeutic planning [84]. In CCKBR-directed strategies, imaging serves as a functional assessment of target suitability by evaluating tumor uptake, receptor accessibility, and tumor-to-organ contrast before therapy [8]. Consequently, imaging identifies patients most likely to benefit from receptor-mediated delivery while reducing the risk of ineffective treatment in cases with insufficient target engagement.

Clinical experience has demonstrated that receptor expression alone is insufficient to predict therapeutic success. Instead, treatment feasibility depends on the combined influence of uptake intensity, lesion distribution, and contrast relative to physiologic sink organs. CCKBR-targeted PET and SPECT imaging provide whole-body evaluation of these parameters and support selection of patients with favorable biodistribution profiles [7,10,74]. In addition, pre-therapeutic imaging enables individualized dosimetry, estimation of radiation exposure, and assessment of potential toxicity risks before radionuclide therapy [85,86]. Representative clinical studies and their reported imaging performance are summarized in Table 1.

Beyond patient selection, molecular imaging plays an important role in the development of next-generation CCKBR-targeted therapies. Imaging enables in vivo evaluation of radiopharmaceutical engineering strategies designed to improve biodistribution and tumor retention [34,87]. Serial imaging may also facilitate longitudinal assessment of tracer distribution and treatment-related changes over time. Collectively, current evidence supports CCKBR-targeted imaging as a critical bridge between receptor biology and therapeutic implementation, forming the foundation of the theranostic pairing paradigm and future receptor-guided precision oncology strategies [8].

Therapeutic development has primarily focused on radiolabeled minigastrin analogues, particularly PP-F11N and CP04, which have demonstrated favorable tumor retention and therapeutic efficacy in CCKBR-expressing tumors. Progressive optimization of peptide structure, linker composition, and radiometal selection has substantially improved metabolic stability and tumor targeting compared with earlier gastrin derivatives. Nevertheless, renal uptake remains the principal dose-limiting factor for therapeutic application, emphasizing the need for continued ligand engineering and nephroprotective strategies. The recent introduction of α-emitting radionuclides, including ^225^Ac, has further expanded the therapeutic potential of CCKBR-targeted radiopharmaceuticals by enhancing tumor cell killing while maintaining receptor specificity. Collectively, these studies support the feasibility of CCKBR-directed peptide receptor radionuclide therapy (PRRT) and provide a strong translational framework for future clinical trials and personalized theranostic strategies.

Overall, these therapeutic studies illustrate the evolution of CCKBR-targeted radiopharmaceuticals from proof-of-concept compounds to clinically translatable theranostic agents, highlighting the importance of integrating receptor biology, radiochemistry, dosimetry, and patient selection in the development of next-generation precision oncology. From a theranostic perspective, clinically investigated CCKBR-targeted diagnostic approaches have primarily involved ^111^In-labeled SPECT and ^68^Ga-labeled PET tracers, whereas therapeutic development has mainly focused on ^177^Lu-labeled analogues. Although these studies support the feasibility of CCKBR-directed imaging and radionuclide therapy, standardized diagnostic–therapeutic pairing for treatment selection has not yet been established.

### 4.4. Pharmacokinetics and Tumor-to-Organ Contrast

The clinical utility of CCKBR-targeted imaging depends not only on receptor affinity but also on pharmacokinetic properties that determine tumor retention, tissue distribution, and physiologic clearance. Following systemic administration, peptide tracers bind CCKBR-expressing tumor cells and undergo receptor-mediated internalization, resulting in intracellular accumulation and prolonged signal retention [19,74]. This internalization process contributes to sustained tumor visualization and distinguishes CCKBR-targeted imaging from non-internalizing receptor systems [88].

Tumor retention must be interpreted in the context of physiologic clearance pathways. Most mini-gastrin–based tracers undergo predominant renal elimination, whereas gastric uptake reflects physiologic CCKBR expression within the fundic mucosa [32,89]. As circulating tracer is progressively cleared from non-target tissues, receptor-positive tumors may maintain prolonged signal retention, resulting in improved tumor-to-background contrast over time [10,89]. Consequently, tumor-to-kidney and tumor-to-stomach uptake ratios have emerged as practical imaging biomarkers for assessing target suitability and potential therapeutic retention [85,90].

Pharmacokinetic analysis also plays an important role in theranostic development and safety assessment. Molecular imaging enables in vivo evaluation of peptide-engineering strategies designed to optimize biodistribution and tumor uptake, while time–activity curve analysis supports estimation of organ radiation exposure and individualized dosimetry [91,92,93]. Collectively, imaging pharmacokinetics provide a framework for balancing tumor targeting, normal-organ exposure, and therapeutic feasibility, thereby supporting the clinical translation of CCKBR-directed theranostic strategies.

## 5. Therapeutic Applications of CCKBR-Targeted Therapies

The ability of CCKBR to undergo ligand-induced internalization makes it an attractive target for receptor-directed therapy. Following successful development of CCKBR-targeted imaging agents, therapeutic strategies have increasingly focused on exploiting receptor-mediated intracellular delivery to enhance tumor retention and treatment efficacy [13,19]. These approaches primarily include peptide receptor radionuclide therapy (PRRT), which delivers targeted radiation through radiolabeled peptides and promotes intracellular retention within receptor-expressing tumor cells [24].

Clinical translation of these therapies requires optimization of tumor uptake while minimizing exposure to physiologic uptake organs, particularly the stomach and kidneys [91]. The clinical development pathway of CCKBR-targeted PRRT closely parallels the evolution of SSTR-directed therapy represented by ^177^Lu-DOTATATE. However, unlike the latter, CCKBR-directed radionuclide therapy remains at an earlier stage of clinical translation, with limited prospective clinical evidence and no approved therapeutic agent to date. Advances in radiochemistry, peptide stabilization, and pharmacokinetic engineering have substantially improved tumor-to-organ contrast and expanded the therapeutic potential of CCKBR-targeted platforms. This chapter reviews current developments in CCKBR-directed radionuclide therapy and strategies aimed at improving therapeutic efficacy and safety. Together, these advances highlight the potential of CCKBR-targeted therapies as an emerging component of precision oncology.

### 5.1. CCKBR-Targeted Radionuclide Therapy

CCKBR-targeted radionuclide therapy has primarily been developed using mini-gastrin–based peptide scaffolds that retain high-affinity receptor binding and efficient receptor-mediated internalization [94,95]. Early preclinical studies demonstrated selective accumulation of radiolabeled gastrin analogs within receptor-positive tumors, establishing the feasibility of targeted radionuclide delivery [81,83]. Among currently available therapeutic radionuclides, ^177^Lu-labeled peptide constructs represent the most advanced platform. The favorable physical properties of ^177^Lu, including medium-range β-particle emission and concurrent γ-photon emission for post-therapy imaging, support both therapeutic efficacy and individualized dosimetry assessment [83,96]. Consequently, ^177^Lu-labeled mini-gastrin analogs have become the leading candidates for CCKBR-directed peptide receptor radionuclide therapy (PRRT).

Alternative radionuclides have also been investigated to address different therapeutic requirements. 90Y provides higher β-particle energy and longer tissue penetration, potentially benefiting larger or spatially heterogeneous tumors [97]. Emerging radionuclides such as ^161^Tb may enhance cytotoxicity through the additional emission of Auger electrons, whereas ^67^Cu offers the potential for matched ^64^Cu/^67^Cu theranostic applications using chemically identical chelation systems [97,98]. Therefore, radionuclide selection should be guided by tumor size and heterogeneity, receptor density, ligand pharmacokinetics, desired particle range, imaging/dosimetry requirements, and radionuclide availability rather than by a single universally optimal isotope. Collectively, current evidence supports CCKBR-targeted PRRT as the most advanced therapeutic application of CCKBR-directed theranostics. Continued optimization of radionuclide selection, peptide design, and treatment dosimetry is expected to further improve therapeutic efficacy and expand clinical applicability [94,97]. ^177^Lu post-treatment SPECT/CT is already supported by dedicated clinical guidelines, whereas ^161^Tb and ^67^Cu remain much less widely available clinically. Nuclear half-life and decay properties can be cross-checked against evaluated NNDC/ENSDF data. The key physical, dosimetric, and clinical characteristics of therapeutic radionuclides relevant to CCKBR-targeted applications are summarized in Table 2.

Radionuclide selection should therefore be guided by tumor size, spatial heterogeneity, pharmacokinetics, and the need for post-treatment imaging rather than by a single universally optimal isotope. ^177^Lu currently offers the most practical balance between tissue range, imaging capability, dosimetry, and clinical availability, whereas ^90^Y may favor larger or heterogeneous lesions. ^161^Tb and ^67^Cu provide attractive next-generation options because of their enhanced short-range energy deposition and matched theranostic potential, respectively, but remain investigational for CCKBR-targeted applications.

### 5.2. Preclinical and Early Clinical Evidence

Preclinical studies have provided the strongest evidence supporting the feasibility of CCKBR-targeted radionuclide therapy. In receptor-positive tumor models, radiolabeled mini-gastrin analogues demonstrated efficient tumor uptake, prolonged retention, and antitumor activity, including tumor growth delay and partial regression in selected models [28]. Preclinical studies have also identified renal and gastric exposure as important safety considerations and demonstrated that peptide engineering and amino acid co-infusion can reduce renal retention while preserving tumor targeting [99].

In contrast, clinical evidence remains limited and is derived mainly from early-phase studies. The prospective phase 0a LUMED study evaluated ^177^Lu-PP-F11N in six patients with advanced MTC, primarily demonstrating tumor targeting, safety, and human dosimetry rather than therapeutic efficacy [95]. In addition, the multicenter Phase I GRAN-T-MTC study evaluated ^111^In-CP04 in patients with progressive or metastatic MTC, focusing on safety, biodistribution, dosimetry, and diagnostic performance [100]. Thus, current clinical evidence supports the feasibility and translational potential, rather than established clinical efficacy, of CCKBR-targeted radionuclide therapy. Representative preclinical and clinical studies of CCKBR-targeted radioligand therapy are summarized in Table 3.

### 5.3. Dose-Limiting Toxicities and Mitigation Strategies

The clinical success of CCKBR-targeted radionuclide therapy depends on achieving sufficient tumor irradiation while limiting exposure to normal tissues. The kidneys and gastric mucosa represent the principal dose-limiting organs; however, the mechanisms underlying tracer accumulation differ substantially between these compartments [13,33,101].

Renal radiation exposure is largely driven by peptide reabsorption within proximal tubular epithelial cells rather than receptor-specific binding [102]. Consequently, renal uptake can be reduced through pharmacokinetic interventions, including amino acid co-infusion, peptide modification, and linker optimization, all of which have demonstrated the ability to decrease renal absorbed dose while preserving tumor targeting [86,103,104].

In contrast, gastric uptake reflects physiologic CCKBR expression within the fundic mucosa and therefore results from genuine receptor-mediated binding [10,67,68]. Because gastric accumulation cannot be readily prevented through competitive inhibition strategies, management relies primarily on quantitative imaging, individualized dosimetry, and activity optimization [10,67,89]. Careful activity optimization and individualized treatment planning may further reduce the risk of gastrointestinal toxicity while maintaining therapeutic efficacy [12].

Collectively, effective management of renal and gastric exposure remains essential for maximizing the therapeutic index of CCKBR-targeted radionuclide therapy. Continued advances in peptide engineering, dosimetry, and treatment planning are expected to further improve safety while enabling delivery of higher tumor-absorbed doses.

### 5.4. Mechanisms and Clinical Rationale of CCKBR-Targeted Therapy

The antitumor activity of CCKBR-targeted radionuclide therapy is primarily driven by receptor-mediated internalization and prolonged intracellular retention of therapeutic isotopes [7,13,19]. Following ligand binding, radiolabeled peptides accumulate within receptor-expressing tumor cells, enabling sustained radiation delivery and localized DNA damage [105]. In addition to direct cytotoxicity, β-emitting radionuclides may generate cross-fire effects that extend irradiation to adjacent tumor cells with lower receptor expression, partially compensating for intratumoral heterogeneity [106].

Beyond direct tumor-cell killing, targeted radionuclide therapy may influence the tumor microenvironment through induction of oxidative stress, disruption of stromal signaling, and modulation of immune responses [107,108]. These effects may be particularly relevant in tumors with dense stromal architecture, such as pancreatic cancer, where conventional drug delivery remains challenging [109].

The clinical rationale for CCKBR-targeted therapy is closely linked to the biological dependence of selected tumors on gastrin-responsive signaling pathways and receptor-mediated uptake mechanisms [8,51,109]. Early clinical observations demonstrating disease stabilization and symptomatic improvement in heavily pretreated patients support the therapeutic potential of this approach [10,12]. Despite encouraging results, important challenges remain, including receptor heterogeneity, optimization of treatment schedules, and expansion into epithelial malignancies beyond traditional neuroendocrine indications [110,111,112]. Continued integration of molecular imaging, peptide engineering, and individualized dosimetry is expected to further enhance the clinical utility of CCKBR-targeted therapies.

## 6. Molecular Engineering of CCKBR Theranostics

Not all CCKBR-targeted approaches have translated into improved in vivo performance. Early mini-gastrin analogues were frequently limited by rapid enzymatic degradation, short circulation time, and substantial renal retention, which reduced tumor-to-organ contrast and therapeutic index [113]. Likewise, strategies designed to prolong systemic exposure, such as albumin binding, may increase tumor retention but can also delay clearance from non-target tissues when binding is excessive [8]. Some ligand modifications have therefore improved one pharmacokinetic property at the expense of another. These less successful experiences highlight the need to balance receptor affinity, metabolic stability, tumor retention, and normal-organ exposure rather than optimizing any single parameter in isolation.

The development of effective CCKBR-targeted theranostics relies not only on receptor expression but also on molecular designs that optimize tumor uptake, metabolic stability, and biodistribution. Although early mini-gastrin–based ligands demonstrated the feasibility of receptor-directed targeting, their clinical utility was limited by rapid enzymatic degradation and unfavorable pharmacokinetic properties [10,67]. Consequently, substantial efforts have focused on engineering peptide-based platforms with improved stability, tumor retention, and therapeutic index.

Current development strategies integrate peptide scaffold optimization, linker design, chelator chemistry, and pharmacokinetic modulation into a unified engineering framework [13,114]. Through systematic modification of peptide structure and molecular properties, investigators aim to enhance receptor targeting while reducing exposure to normal tissues. These advances have expanded the versatility of CCKBR-directed agents for both molecular imaging and therapeutic applications.

This chapter reviews the major molecular-engineering approaches that have shaped CCKBR theranostic development, with emphasis on scaffold evolution, metabolic stabilization, pharmacokinetic optimization, and structure–activity relationships that influence clinical performance (Figure 4).

### 6.1. Peptide Evolution of Mini-Gastrin-Based Ligands and Evolution

CCKBR-targeted theranostics originated from endogenous gastrin and cholecystokinin peptides, whose conserved C-terminal Trp-Met-Asp-Phe-NH_2_ pharmacophore medilates high-affinity receptor recognition and internalization [115,116]. Early mini-gastrin analogs established the feasibility of receptor-directed targeting but were limited by rapid enzymatic degradation and unfavorable pharmacokinetics, prompting extensive efforts to improve ligand stability and tumor retention [10,67].

Subsequent ligand optimization focused on modifying peptide regions outside the core pharmacophore while preserving receptor binding and intracellular delivery. Common peptide-engineering approaches to improve metabolic stability include amino acid substitution, incorporation of non-natural or D-amino acids, backbone modification, and sequence truncation [117,118,119]. Rather than maximizing receptor affinity alone, modern design strategies increasingly emphasize efficient receptor-mediated internalization and prolonged intracellular retention, both of which are critical determinants of therapeutic performance.

Scaffold evolution has further incorporated modifications in peptide length, charge distribution, hydrophilicity, and chelator design to optimize receptor accessibility and biodistribution [13,114]. Collectively, these iterative engineering efforts have progressively improved the pharmacological properties of mini-gastrin analogues and established the foundation for the development of current-generation CCKBR-targeted radiopharmaceuticals, while continued optimization remains an active area of research suitable for both molecular imaging and therapeutic applications, providing the foundation for next-generation CCKBR-directed theranostic platforms (Table 4).

### 6.2. Current Strategies for Pharmacokinetic Optimization

Although receptor binding and internalization determine target engagement, systemic pharmacokinetics ultimately govern tumor exposure and therapeutic efficacy in vivo [120]. Despite these substantial advances, several pharmacokinetic limitations continue to restrict the clinical performance of current CCKBR-targeted ligands. These remaining challenges have motivated further optimization strategies aimed at improving metabolic stability, tumor retention, and kidney clearance [8,121].

One of the most effective approaches involves modulation of plasma protein interactions through incorporation of albumin-binding motifs. Reversible association with serum albumin extends circulation half-life, increases opportunities for tumor receptor engagement, and can improve tumor-to-kidney uptake ratios [8,122,123]. However, excessive albumin binding may delay clearance from non-target tissues, requiring careful optimization of binding strength and molecular design.

Linker architecture represents an additional determinant of biodistribution and tumor retention. Modifications in linker composition, charge distribution, hydrophilicity, and molecular flexibility influence metabolic stability, tissue distribution, and receptor accessibility [124,125,126]. Together, optimization of albumin binding, linker design, and molecular charge has enabled the development of CCKBR-targeted ligands with improved pharmacokinetic profiles, prolonged tumor residence, and enhanced therapeutic index [8,125].

### 6.3. Chelator and Radiometal Engineering

Chelator selection is a critical determinant of the biological performance of CCKBR-targeted theranostic agents. Beyond radiometal coordination, chelators influence molecular charge, hydrophilicity, conformational flexibility, and complex stability, all of which affect tumor uptake, biodistribution, and normal-organ exposure [13]. Consequently, chelator design has become an integral component of ligand optimization rather than a purely radiochemical consideration.

Macrocyclic chelators such as DOTA remain the most widely used platforms because of their high thermodynamic stability and compatibility with multiple diagnostic and therapeutic radionuclides, including ^68^Ga and ^177^Lu [127]. Alternative systems, including NOTA and NODAGA, offer distinct coordination properties that may improve labeling efficiency and alter biodistribution profiles [30,128]. In parallel, radiometal selection also contributes to pharmacologic behavior through differences in decay characteristics, coordination chemistry, and in vivo stability. For example, ^68^Ga, ^177^Lu, and copper-based theranostic pairs may exhibit different clearance patterns and organ uptake profiles despite sharing identical peptide scaffolds [129].

The spatial relationship between the chelator and receptor-binding domain further influences receptor accessibility, metabolic stability, and pharmacokinetic behavior [130]. Collectively, coordinated optimization of chelator systems, radiometal selection, and ligand architecture has become an essential strategy for improving tumor contrast, reducing off-target exposure, and enhancing the therapeutic index of CCKBR-targeted theranostic agents [8].

## 7. Future Perspectives and Clinical Translation

The continued development of CCKBR-directed theranostics will depend on successful integration of advances in molecular imaging, radionuclide therapy, and ligand engineering into clinically scalable treatment platforms. Although substantial progress has been achieved in tracer development, pharmacokinetic optimization, and early therapeutic evaluation, broader clinical implementation will require standardized imaging protocols, robust dosimetry frameworks, and prospective multicenter validation studies.

Emerging technologies, including advanced quantitative imaging analytics, next-generation radionuclide pairs, and data-driven approaches for patient stratification, may further improve treatment selection and therapeutic precision. At the same time, continued advances in peptide engineering and molecular design are expected to enhance tumor targeting while reducing normal-organ exposure.

Ultimately, the successful translation of CCKBR-targeted theranostics will depend not only on technological innovation but also on coordinated clinical infrastructure, regulatory harmonization, and sustainable radionuclide supply networks. Collectively, these priorities define a translational roadmap for advancing CCKBR-targeted theranostics from early clinical investigation toward standardized and scalable clinical implementation (Figure 5). Future development of CCKBR-targeted theranostics is expected to follow a translational pathway similar to that established for SSTR-directed theranostics, including standardized imaging, prospective dosimetry, multicenter clinical trials, and regulatory approval.

### 7.1. Clinical Translation and Theranostic Infrastructure

The successful clinical implementation of CCKBR-targeted theranostics requires more than advances in molecular design alone. Sustainable translation depends on coordinated infrastructure encompassing radionuclide production, peptide manufacturing, radiolabeling, quality control, and clinical delivery. Standardized production pipelines supported by Good Manufacturing Practice (GMP) facilities and harmonized quality assurance procedures will be essential for ensuring reproducibility and scalability across institutions.

Multi-institutional collaboration represents another key component of clinical development. Shared imaging registries, dosimetry databases, and prospective clinical studies may facilitate validation of patient-selection criteria, treatment protocols, and safety outcomes, particularly for relatively uncommon receptor-positive malignancies. Harmonization of imaging acquisition and quantitative analysis will further improve comparability of clinical data across centers.

Reliable radionuclide supply chains and regulatory support are equally important for long-term implementation. Integration of isotope production, radiopharmaceutical manufacturing, and distribution networks can enhance treatment accessibility while reducing vulnerability to supply interruptions. Together, these efforts may facilitate the transition of CCKBR-targeted theranostics from a promising investigational platform to a scalable and sustainable component of precision oncology.

Importantly, these translational barriers are not unique to CCKBR-targeted radiopharmaceuticals. Similar challenges were encountered during the clinical development of established PRRT platforms, particularly SSTR-targeted therapy with ^177^Lu-DOTATATE. Its successful translation was facilitated by standardized radiopharmaceutical production under GMP conditions, centralized or qualified manufacturing networks, harmonized imaging and dosimetry protocols, multicenter prospective trials, and clearly defined regulatory pathways. Routine use was further supported by standardized patient-selection criteria based on receptor imaging and by established procedures for radiation safety, treatment delivery, and post-treatment monitoring. These developments provide a practical framework for CCKBR-targeted theranostics. However, CCKBR-directed agents face additional challenges, including the need for further ligand optimization, reduction in renal uptake, validation of receptor-expression thresholds for patient selection, and generation of sufficiently large prospective clinical datasets. Therefore, progress toward clinical implementation will require not only improved radiopharmaceutical performance but also early integration of scalable GMP manufacturing, standardized imaging and dosimetry, multicenter clinical validation, and regulatory planning. Lessons learned from approved PRRT agents may substantially accelerate this translational pathway rather than requiring CCKBR-targeted therapies to establish an entirely new clinical infrastructure.

### 7.2. Emerging Technologies and Future Clinical Translation

Emerging technologies are expected to play an increasingly important role in the future development of CCKBR-targeted theranostics. Artificial intelligence (AI) and advanced quantitative imaging analytics may enable automated lesion segmentation, quantitative receptor mapping, and prediction of therapeutic response from complex imaging dataset [37]. Integration of imaging, dosimetry, and clinical outcome data could further support more accurate patient stratification and individualized treatment planning.

At the same time, next-generation theranostic radionuclides are expanding the versatility of receptor-directed therapy. Emerging isotope pairs, including ^64^Cu/^67^Cu, ^44^Sc/^47^Sc, and terbium-based systems, offer opportunities for improved imaging performance and more tailored radiation delivery according to tumor size, receptor density, and biological heterogeneity [131,132]. These advances may further refine the precision of theranostic workflows and broaden the range of clinically applicable treatment strategies [133].

Beyond technological innovation, successful clinical adoption will require scalable manufacturing, reliable radionuclide supply chains, and regulatory harmonization. Strategic collaboration among academic institutions, healthcare systems, radionuclide producers, and industry partners will be essential for translating promising research into sustainable clinical practice. Together, advances in artificial intelligence, next-generation radionuclides, and clinical infrastructure are expected to facilitate the transition of CCKBR-targeted theranostics from a promising investigational platform to an established component of precision oncology.

### 7.3. Artificial Intelligence in CCKBR-Targeted Theranostics

Recent advances in artificial intelligence (AI) have created new opportunities to further enhance the clinical implementation of CCKBR-targeted theranostics [134,135,136]. Beyond conventional image interpretation, AI-based algorithms have demonstrated increasing value in lesion detection, automated segmentation, quantitative image analysis, and radiomics-based feature extraction from PET and SPECT images. By integrating imaging biomarkers with clinical and pathological information, machine learning models may improve the identification of patients who are most likely to benefit from CCKBR-targeted imaging and radionuclide therapy. Such approaches could be particularly valuable in tumors exhibiting heterogeneous receptor expression, where conventional visual assessment alone may underestimate biologically relevant disease burden. As more multicenter imaging datasets become available, AI-assisted analysis may facilitate standardized image interpretation, improve reproducibility across institutions, and support evidence-based patient selection for precision theranostic applications.

Beyond diagnostic imaging, AI also has considerable potential to optimize the entire theranostic workflow [137,138]. Deep learning models may assist in individualized dosimetry, prediction of treatment response, and estimation of radiation exposure to critical organs such as the kidneys and stomach, thereby supporting personalized treatment planning and toxicity risk assessment. Furthermore, AI-driven computational approaches may accelerate ligand discovery through virtual screening, peptide optimization, and structure–activity relationship analysis, shortening the development cycle of next-generation CCKBR-targeted radiopharmaceuticals. When combined with multimodal data including molecular imaging, genomic profiling, and clinical outcomes, AI may enable adaptive treatment strategies and real-time therapeutic decision-making. Although these applications remain at an early stage for CCKBR-targeted agents, the rapid evolution of AI is expected to become an integral component of future receptor-guided precision oncology, facilitating more accurate diagnosis, optimized radionuclide therapy, and individualized patient management [136]. Taken together, the integration of AI with molecular imaging, radiopharmaceutical development, and personalized dosimetry represents an emerging direction that may substantially accelerate the translation of CCKBR-targeted theranostics from experimental research to routine clinical practice.

## 8. Conclusions

CCKBR-targeted theranostics represent a promising approach that integrates molecular imaging and targeted radionuclide therapy within a receptor-guided precision oncology framework. The tumor-associated expression of CCKBR, together with high-affinity ligand recognition and efficient receptor-mediated internalization, provides a strong biological basis for selective radiopharmaceutical delivery. Advances in mini-gastrin-based ligand design, pharmacokinetic optimization, and radiometal chemistry have progressively improved tumor targeting and reduced off-target exposure. Preclinical studies and early clinical investigations, particularly in medullary thyroid carcinoma, have demonstrated the feasibility of CCKBR-directed imaging and radionuclide therapy, while emerging evidence suggests potential applications in other CCKBR-expressing malignancies.

However, the principal limitation is no longer proof of biological feasibility, but the lack of evidence required to establish clinical utility. Four knowledge gaps are particularly important: the extent and clinical significance of inter- and intratumoral CCKBR heterogeneity; the absence of standardized quantitative imaging criteria for patient selection; insufficient prospective evidence defining therapeutic efficacy, dosimetry, and long-term safety; and the need to determine whether current ligand-engineering strategies can achieve a clinically acceptable therapeutic index, particularly with respect to renal and gastric exposure. Until these questions are addressed, CCKBR-targeted theranostics should be regarded as less clinically established than SSTR- or PSMA-based platforms despite substantial experimental support. Addressing these gaps through prospective, quantitatively designed clinical studies will determine whether CCKBR can progress from a biologically compelling target to a clinically validated theranostic platform.

## Figures and Tables

**Figure 1:**
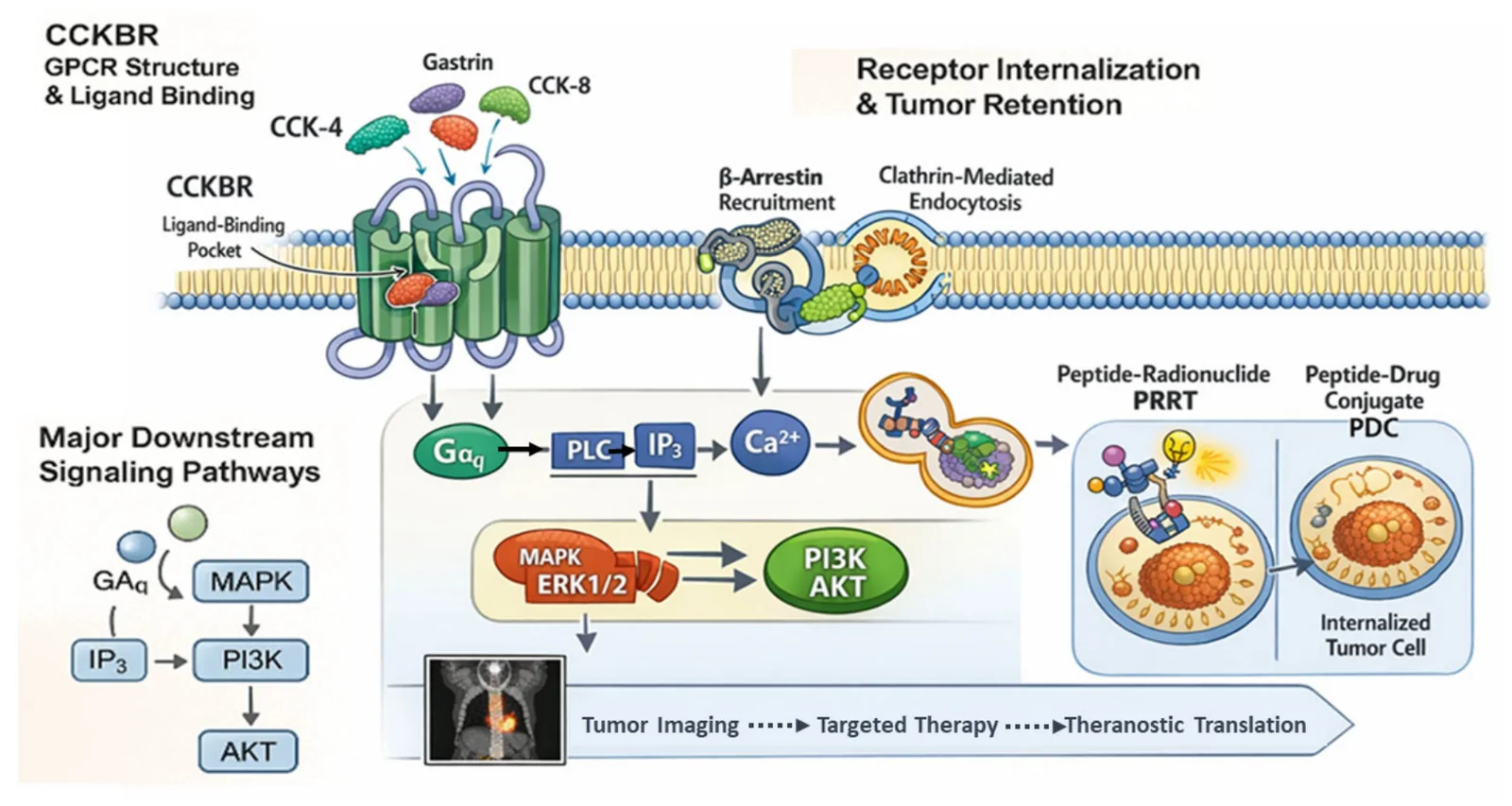
**CCKBR structure, signaling, receptor internalization, and their relevance to theranostic applications.** Binding of gastrin- and CCK-derived ligands to CCKBR activates predominantly Gq-coupled signaling, including the PLC–IP3–Ca^2+^ cascade and downstream MAPK/ERK and PI3K/AKT pathways. Agonist-induced receptor activation also promotes β-arrestin recruitment and clathrin-mediated endocytosis, facilitating intracellular accumulation of receptor-bound ligands. Solid arrows indicate mechanisms that are well established by experimental evidence, including ligand binding, Gq-mediated signaling, β-arrestin recruitment, and receptor internalization. Dashed arrows indicate proposed or translational relationships supported by current preclinical and emerging clinical evidence, particularly the linkage between receptor internalization/tumor retention and improved molecular imaging or therapeutic efficacy. These biological properties collectively provide the mechanistic rationale for CCKBR-targeted imaging, peptide receptor radionuclide therapy (PRRT), and other emerging ligand-directed therapeutic strategies.

**Figure 2:**
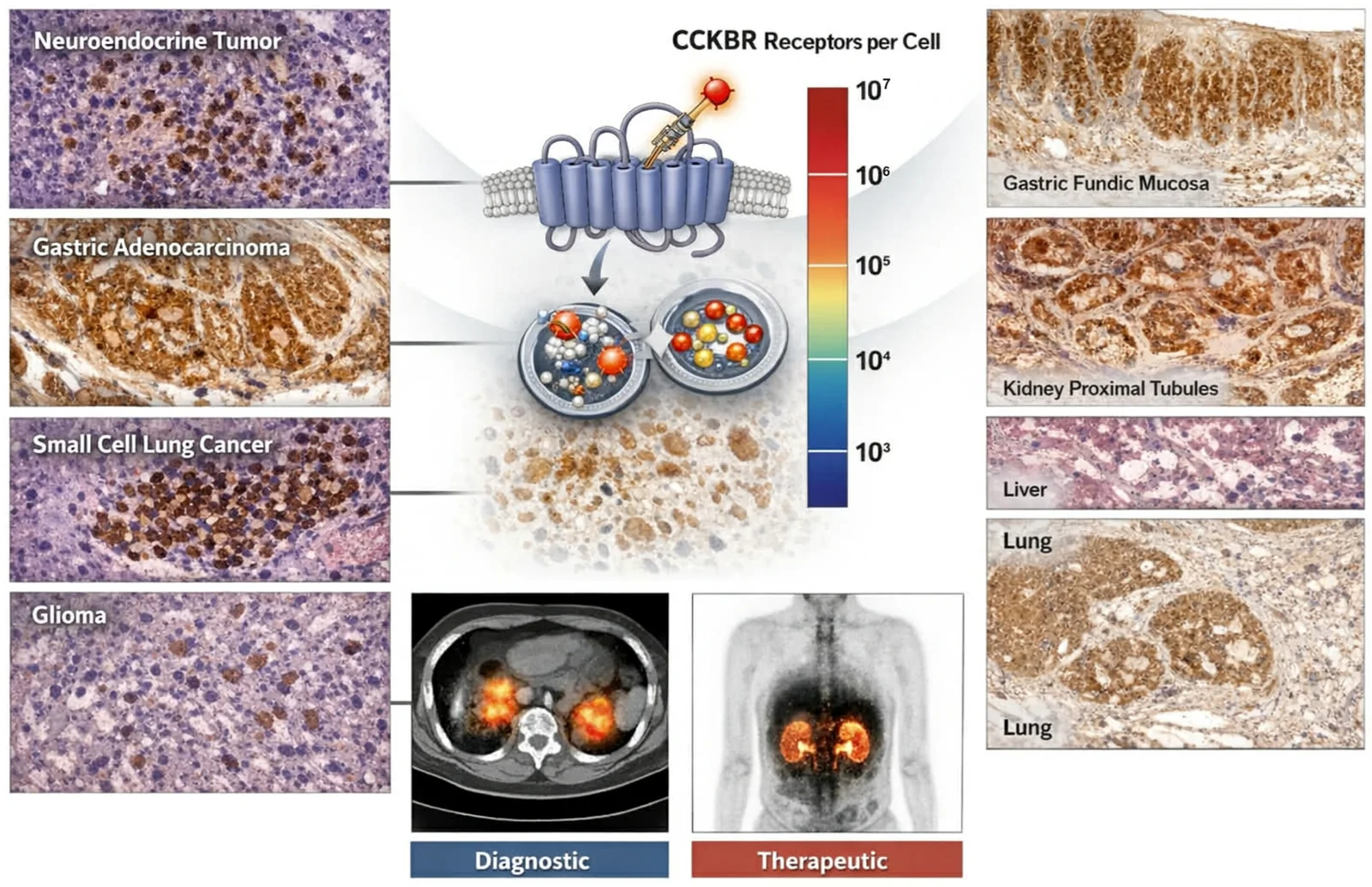
**Conceptual illustration of the tumor–normal tissue expression landscape of CCKBR for theranostic applications.** This AI-assisted schematic was created by the authors based on the published evidence summarized in the manuscript and is intended for conceptual visualization only. It does not represent original quantitative data or data reproduced from a single study. The imaging and therapy panels illustrate the conceptual relationships among receptor expression, target visualization, tumor uptake, and therapeutic response. Colors in the therapy panel indicate relative conceptual differences in therapeutic effect and should not be interpreted as quantitative clinical efficacy data.

**Figure 3:**
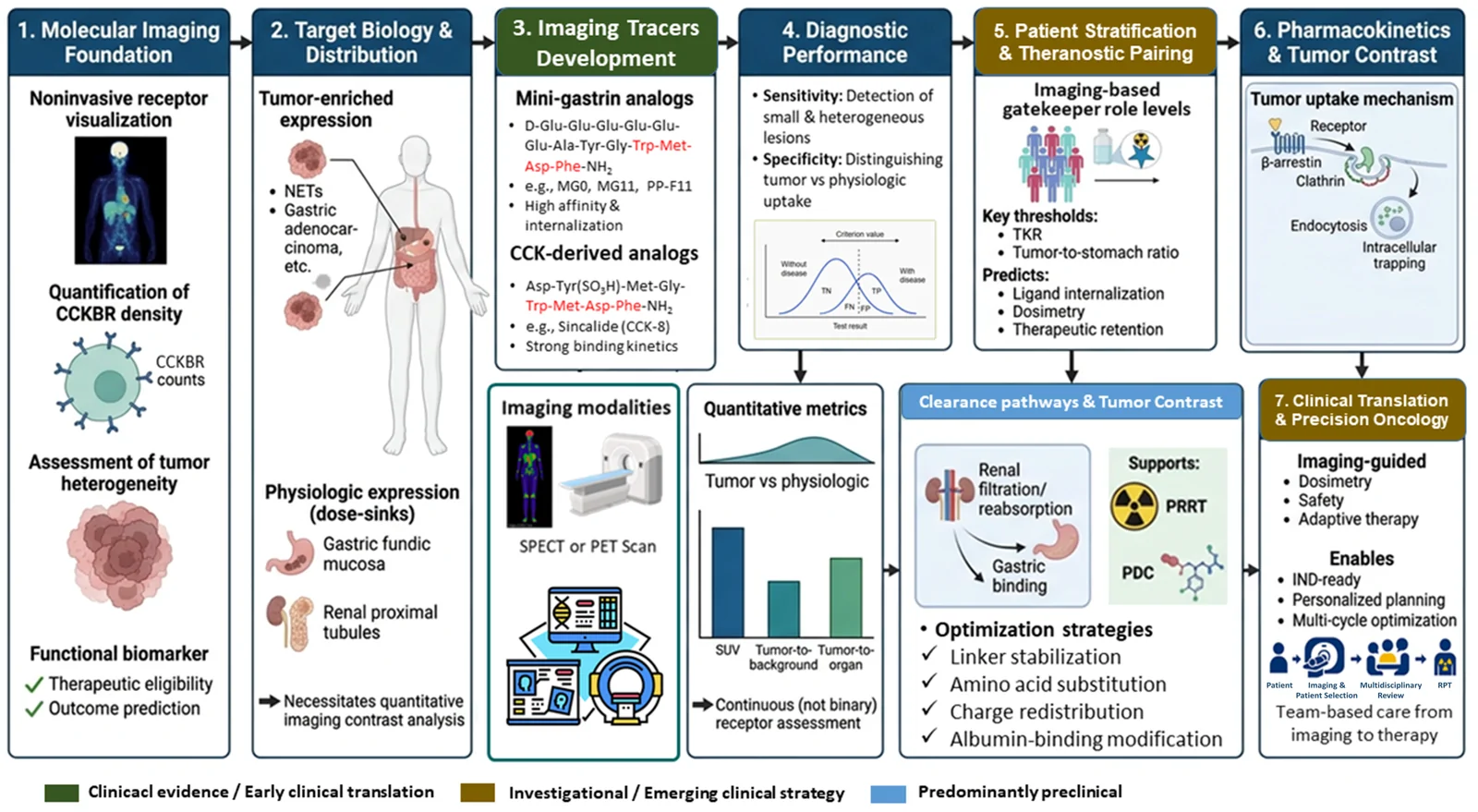
**Translational framework and current development status of CCKBR-targeted theranostics.** Schematic overview of the translational workflow for CCKBR-targeted theranostics, with approaches distinguished according to their current level of clinical development. Clinically investigated approaches include CCKBR-targeted molecular imaging and early imaging-based patient assessment evaluated in human studies, whereas preclinical or investigational approaches include next-generation ligand engineering, pharmacokinetic optimization, emerging radionuclides, advanced dosimetry, and AI-assisted precision theranostic strategies. Although several CCKBR-targeted agents have entered clinical investigation, none has yet achieved the level of routine clinical implementation established for SSTR- or PSMA-directed theranostics.

**Figure 4:**
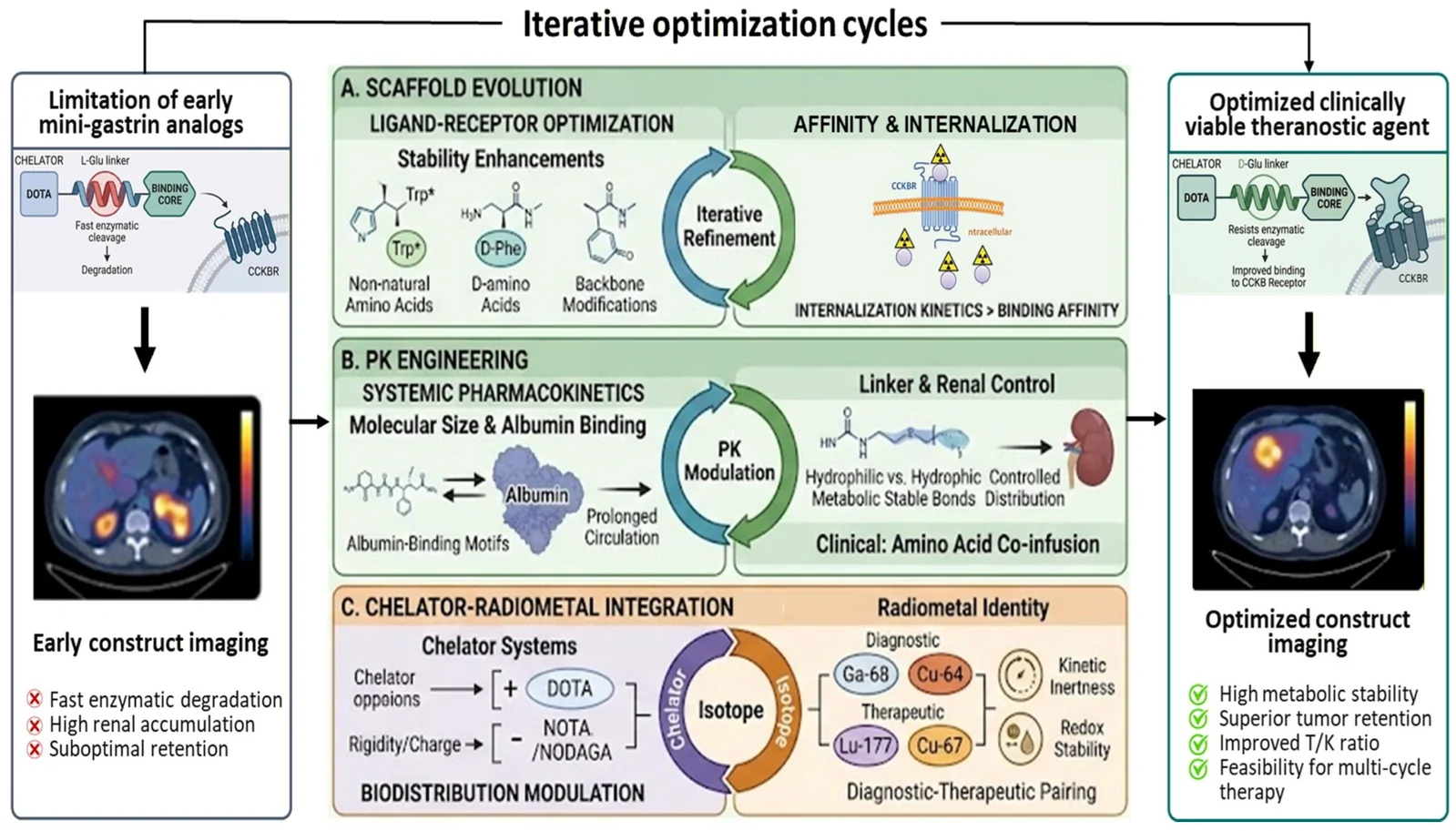
**Optimization strategies for CCKBR-targeted theranostic tracers.** Schematic overview of the major optimization strategies used in CCKBR-targeted tracer development. Scaffold modification improves receptor affinity, stability, and internalization; pharmacokinetic engineering enhances tumor delivery while reducing renal exposure; and chelator–radiometal selection influences biodistribution, imaging performance, and therapeutic efficacy. Together, these approaches expand the therapeutic window of CCKBR-directed imaging and treatment platforms.

**Figure 5:**
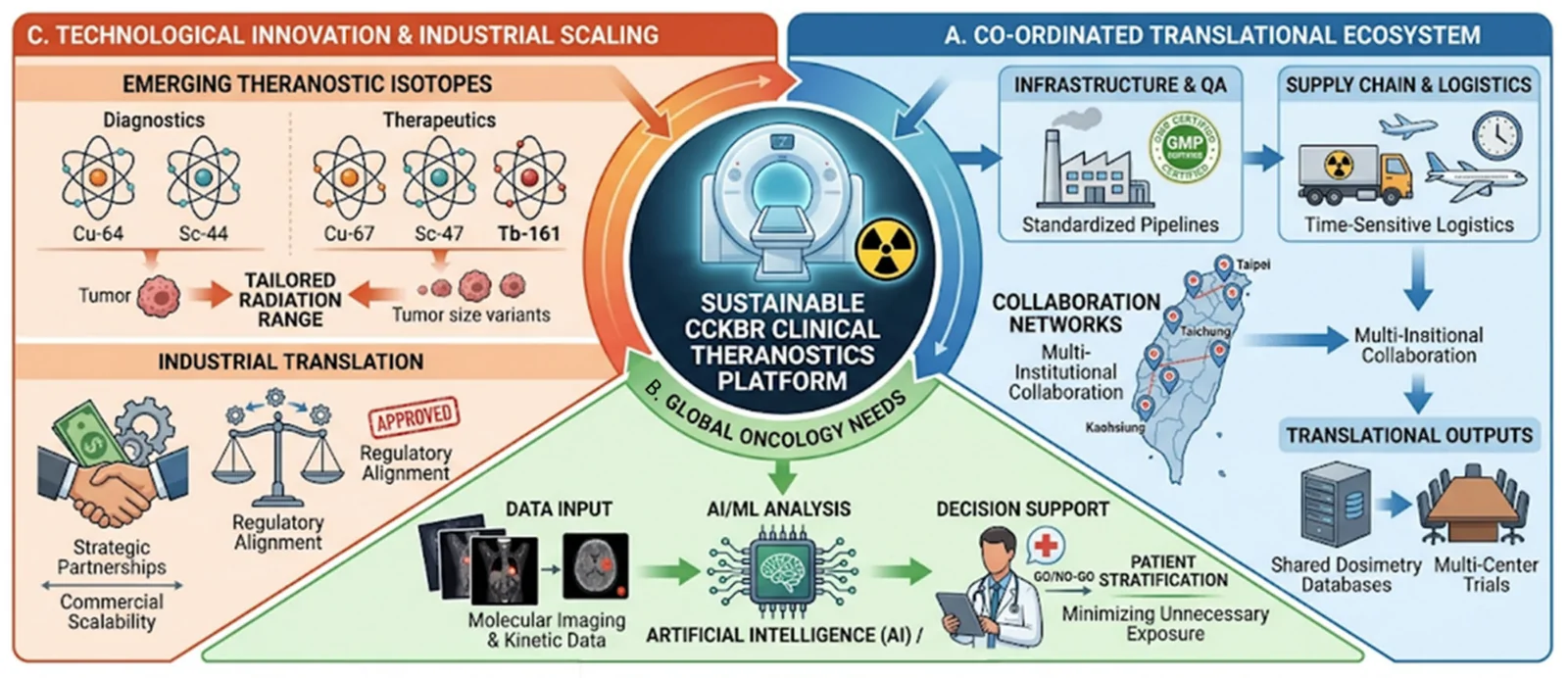
**Roadmap toward sustainable clinical implementation of CCKBR-targeted theranostics.** The figure illustrates an integrated translational framework designed to facilitate the transition of CCKBR-targeted theranostic strategies from experimental research to routine clinical application. Three interdependent pillars are highlighted: (**A**) Co-ordinated Translational Ecosystem, encompassing infrastructure development, quality assurance, regulatory harmonization, and multi-institutional collaboration; (**B**) Precision Medicine and Artificial Intelligence Integration, leveraging quantitative molecular imaging, dosimetry, and data-driven analytics to optimize patient selection and treatment decision-making; and (**C**) Technological Innovation and Industrial Scaling, focusing on next-generation radionuclides, theranostic isotope pairs, commercial manufacturing, and supply-chain sustainability. Together, these elements establish a scalable and resilient precision oncology platform capable of supporting the long-term clinical adoption of CCKBR-directed imaging and radionuclide therapy.

**Table 1:** Representative clinical studies investigating CCKBR-targeted molecular imaging in human cancers. Clinical evidence accumulated over the past decade has established radiolabeled minigastrin analogues as the principal imaging agents for CCKBR-expressing tumors, particularly medullary thyroid carcinoma. Most studies consistently demonstrated favorable tumor visualization with acceptable safety profiles, supporting the feasibility of receptor-specific imaging for lesion detection and patient selection prior to PRRT. More recent investigations have extended CCKBR imaging to additional malignancies, including gastric cancer and small-cell lung cancer, although clinical experience remains limited. Collectively, these studies highlight that imaging performance depends not only on receptor density but also on ligand design, radiometal selection, pharmacokinetic optimization, and tumor retention, thereby providing a translational foundation for the continued development of CCKBR-targeted theranostic agents.

Study	Tumor	N	Radiotracer	Imaging Modality	Detection Rate	SUVmax *	Clinical/Translational Significance
Behe et al.	Medullary thyroid carcinoma (MTC)	8	^111^In-CP04	SPECT/CT	High lesion detection	NR	First clinical demonstration of safe and specific CCKBR imaging in patients with MTC.
Klingler et al.	MTC	8	^111^In-CP04	SPECT/CT	90% of known lesions	NR	Confirmed high tumor uptake and favorable biodistribution, supporting receptor-targeted imaging.
Ocak et al.	MTC	10	^68^Ga-CP04	PET/CT	High sensitivity	8–30	Improved lesion visualization and image resolution compared with conventional scintigraphy.
Persico et al.	Recurrent MTC	10	^68^Ga-DOTA-MGS5	PET/CT	High detection rate	10–35	Demonstrated feasibility of PET-based patient selection for CCKBR-targeted radionuclide therapy.
Various clinical studies	Gastric cancer	Small cohorts	Radiolabeled minigastrin analogues	PET/CT	Variable	NR	Demonstrated heterogeneous CCKBR expression, suggesting potential application in selected patients.
Pilot studies	Small-cell lung cancer (SCLC)	Pilot	CCKBR-targeted tracers	PET/CT	Preliminary positive findings	NR	Supports expansion of CCKBR imaging beyond MTC into other receptor-positive malignancies.

Abbreviations: NR, not reported. * SUVmax values vary among studies because of differences in radiotracers, acquisition protocols, injected activity, and patient populations.

**Table 2:** **Comparison of therapeutic radionuclides for CCKBR-targeted theranostic applications**. ^177^Lu, ^90^Y, ^161^Tb, and ^67^Cu are compared according to their physical half-life, particle range, imaging capability, clinical availability, dosimetric characteristics, and potential applications. These differences may guide radionuclide selection according to tumor size, heterogeneity, pharmacokinetics, and imaging/dosimetry requirements.

Radionuclide	Physical Half-Life	Particle /Range	Imaging Capability	Clinical Availability	Main Dosimetric Feature	Potential CCKBR Application
^177^Lu	6.65 d	β−, short–intermediate range (~1–2 mm mean; max ~2 mm)	γ emission enables SPECT/dosimetry	Established	Favorable for small–medium lesions; practical post-therapy dosimetry	Current reference radionuclide for CCKBR PRRT development
^90^Y	64.1 h	High-energy β−, longer range (max ~11 mm)	Limited; bremsstrahlung/PET possible but less practical	Established	Greater cross-fire effect, but potentially higher normal-tissue dose	Larger or heterogeneous tumors
^161^Tb	6.95 d	β− plus abundant conversion/Auger electrons	γ emissions permit SPECT	Emerging/investigational	Higher short-range energy deposition than ^177^Lu	Small lesions, micrometastases, receptor-heterogeneous disease
^67^Cu	61.8 h	β−, intermediate range	γ emission enables SPECT; matched with ^64^Cu PET	Limited/emerging	Potential true matched ^64^Cu/^67^Cu dosimetry	Matched diagnostic–therapeutic CCKBR platform

**Table 3:** Representative Therapeutic Studies of CCKBR-Targeted Radioligands. Representative preclinical and clinical studies evaluating CCKBR-targeted radionuclide therapy.

Ligand	Radionuclide	Model	Major Findings	Limitation	Clinical/Translational Significance
CP04	^177^Lu	Clinical	High tumor retention	Kidney uptake	Proof-of-concept for PRRT
PP-F11N	^177^Lu	Preclinical/Clinical	Improved metabolic stability	Renal accumulation	One of the most promising next-generation CCKBR ligands
PP-F11N	^225^Ac	Preclinical	Higher therapeutic efficacy	Toxicity requires optimization	Potential α-therapy platform
Minigastrin analogues	^177^Lu	Clinical	Favorable dosimetry	Limited patient number	Supports future multicenter clinical trials
DOTA-minigastrin derivatives	Various radionuclides	Preclinical	Improved tumor-to-kidney ratio	Requires further validation	Rational ligand engineering for theranostics

**Table 4:** **Representative molecular engineering strategies and their functional impact on CCKBR-targeted radiopharmaceuticals.** The table summarizes how major ligand-engineering approaches influence receptor affinity and internalization, serum stability, renal uptake, tumor retention, and overall translational performance. Effects are presented qualitatively because their magnitude may vary among individual ligand constructs and experimental models.

Ligand/Strategy	Representative Modification	Receptor Affinity/Internalization	Serum Stability	Renal Uptake	Tumor Retention	Overall Translational Effect
PP-F11N	N-terminal modification	Maintained/high	Improved	Reduced/optimized	Increased	Improved tumor-to-organ profile
CP04	Pentaglutamate containing scaffold	Preserved	Improved	Renal uptake remains relevant	Favorable	Advanced to clinical evaluation
DOTA-MG11	DOTA conjugation	Preserved	Moderate/limited	Relatively high	Moderate	Enabled radiometal-based imaging/therapy
DOTA-PP-F11N	Amino-acid/scaffold optimization	Maintained	Improved	Reduced	Increased	Improved pharmacokinetics and therapeutic index
Albumin binder analogue	Albumin-binding moiety	Generally preserved	Prolonged systemic exposure	Context-dependent	Increased	Potentially improved therapeutic index

## Data Availability

No new data were created or analyzed in this study. Data sharing is not applicable to this article.

## Abbreviations

The following abbreviations are used in this manuscript:

CCKBR	Cholecystokinin B receptor
DOTA	1,4,7,10-Tetraazacyclododecane-1,4,7,10-Tetraacetic Acid
GEP-NETs	Gastroenteropancreatic neuroendocrine tumors
NOTA	1,4,7-triazacyclononane-1,4,7-triacetic acid
NODAGA	1,4,7-triazacyclononane-1-glutaric acid-4,7-diacetic acid
PET	Positron emission tomography
PRRT	Peptide receptor radionuclide therapy
SPECT	Single-photon emission computed tomography
